# TorsinA: a therapeutic target for ALS?

**Published:** 2014-02

Amyotrophic lateral sclerosis (ALS) is a motor neuron disease that is characterised by progressive muscle weakness. Several proteins, including superoxide dismutase 1 (SOD1), have been linked to the disease. However, the mechanisms underlying the clinical pathology and, crucially, the selective vulnerability of neuron subtypes to degeneration remain poorly understood. It has been suggested that disease-susceptible neurons are more prone to cellular stress in response to protein misfolding in the ER. The lab of Guy and Kim Caldwell explored this possibility using worm (*C. elegans*) models of ALS. Transgenic expression of human mutant SOD1 gives rise to ALS-related phenotypes in *C. elegans*, including the induction of ER stress and locomotive behavioural defects. The authors demonstrate that these phenotypes can be attenuated by increased expression of torsinA, a chaperone-like protein. These findings provide credence to the hypothesis that ER stress is a key contributor to ALS pathogenesis, and suggest that small-molecule enhancers of torsinA could provide new therapeutic avenues for the disease. Page 233

